# Effects of Individualized Anemia Therapy on Hemoglobin Stability: A Randomized Controlled Pilot Trial in Patients on Hemodialysis

**DOI:** 10.2215/CJN.0000000000000488

**Published:** 2024-06-11

**Authors:** Doris H. Fuertinger, Lin-Chun Wang, David J. Jörg, Lemuel Rivera Fuentes, Xiaoling Ye, Sabrina Casper, Hanjie Zhang, Ariella Mermelstein, Alhaji Cherif, Kevin Ho, Jochen G. Raimann, Lela Tisdale, Peter Kotanko, Stephan Thijssen

**Affiliations:** 1 Fresenius Medical Care Deutschland GmbH, Bad Homburg, Germany; 2 Renal Research Institute, New York, New York; 3 Fresenius Medical Care North America, Waltham, Massachusetts; 4 Department of Medicine, Icahn school of Medicine at Mount Sinai, New York, New York

**Keywords:** anemia, erythropoietin, hemodialysis, randomized controlled trials

## Abstract

**Key Points:**

- We conducted a randomized controlled pilot trial in patients on hemodialysis using a physiology-based individualized anemia therapy assistance software.
- Patients in the group receiving erythropoiesis-stimulating agent dose recommendations from the novel software showed improvement in hemoglobin stability and erythropoiesis-stimulating agent utilization.

**Background:**

Anemia is common among patients on hemodialysis. Maintaining stable hemoglobin levels within predefined target levels can be challenging, particularly in patients with frequent hemoglobin fluctuations both above and below the desired targets. We conducted a multicenter, randomized controlled trial comparing our anemia therapy assistance software against a standard population-based anemia treatment protocol. We hypothesized that personalized dosing of erythropoiesis-stimulating agents (ESAs) improves hemoglobin target attainment.

**Methods:**

Ninety-six patients undergoing hemodialysis and receiving methoxy polyethylene glycol-epoetin beta were randomized 1:1 to the intervention group (personalized ESA dose recommendations computed by the software) or the standard-of-care group for 26 weeks. The therapy assistance software combined a physiology-based mathematical model and a model predictive controller designed to stabilize hemoglobin levels within a tight target range (10–11 g/dl). The primary outcome measure was the percentage of hemoglobin measurements within the target. Secondary outcome measures included measures of hemoglobin variability and ESA utilization.

**Results:**

The intervention group showed an improved median percentage of hemoglobin measurements within target at 47% (interquartile range, 39–58), with a 10% point median difference between the two groups (95% confidence interval, 3 to 16; *P* = 0.008). The odds ratio of being within the hemoglobin target in the standard-of-care group compared with the group receiving the personalized ESA recommendations was 0.68 (95% confidence interval, 0.51 to 0.92). The variability of hemoglobin levels decreased in the intervention group, with the percentage of patients experiencing fluctuating hemoglobin levels being 45% versus 82% in the standard-of-care group. ESA usage was reduced by approximately 25% in the intervention group.

**Conclusions:**

Our results demonstrated an improved hemoglobin target attainment and variability by using personalized ESA recommendations using the physiology-based anemia therapy assistance software.

**Clinical Trial registration number:**

NCT04360902.

## Visual Abstract

**Figure:**
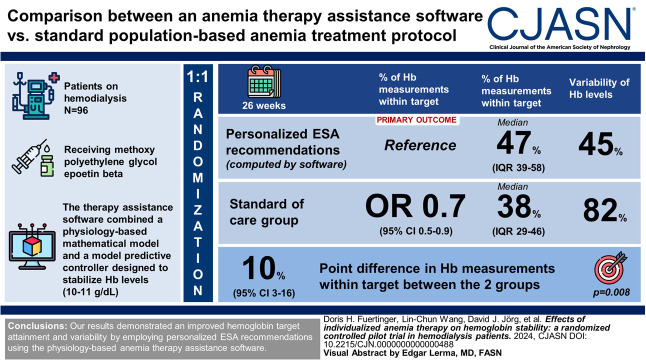

## Introduction

Anemia is common in patients with CKD, particularly in those receiving dialytic KRT, such as hemodialysis. Per the 2022 United States Renal Data System Annual Data Report (“Clinical Indicators and Preventive Care”), 78.2% of patients on hemodialysis received erythropoiesis-stimulating agents (ESAs). Furthermore, among this patient population, methoxy polyethylene glycol-epoetin beta use increased from 24.1% in 2016 to 39.3% in 2020.^1^

A hemoglobin target range of 10–11 or 10–11.5 g/dl is suggested by clinical guidelines, such as Kidney Disease Outcomes Quality Initiative (2007), Kidney Disease Improving Global Outcomes (2012), and US Food and Drug Administration (2011) recommendations.^2^ An increased risk of adverse clinical outcomes (vascular access thrombosis, thromboembolic events, composite cardiovascular events including nonfatal myocardial infarctions, strokes) and mortality was reported for high hemoglobin targets that significantly exceed the currently recommended hemoglobin target ranges or for observed levels falling below them (fatigue, reduced physical performance and quality of life, left ventricular dilation).^3–12^ Randomized trials have highlighted the differences in outcomes associated with the *targeting* of higher hemoglobin levels versus those associated with comparable hemoglobin levels in observational studies.^3,9,12–16^

Achieving and maintaining target hemoglobin levels is challenging because of biological heterogeneity,^17^ a nonlinear dose-response relationship, and time delays between hemoglobin measurement, subsequent ESA administration, and hemoglobin response.^18^ In patients on hemodialysis, hemoglobin levels frequently vary above and below set targets within short time intervals even if mean hemoglobin levels remain within the target range.^3^ Importantly, these frequent hemoglobin excursions affect most patients.^17,19^ The United States Renal Data System and other large longitudinal population studies have shown an association between hemoglobin variability and patient morbidity ^20–22^ and mortality risk.^6,23^ A European study reported a significantly higher likelihood of hospitalization but not of mortality.^22^

Various tools have been introduced over the years to support physicians with anemia management. Treatment protocols are frequently used and considered the standard of care in many dialysis clinics. In addition, software tools, many of which apply machine learning techniques, have been developed and implemented in clinical practice.^24–28^ A shortcoming of these methods is their inherent lack of interpretability of the underlying decision-making process that prevents the clinician from understanding individual ESA dose recommendations. Therefore, we have developed an anemia therapy assistance system that builds on established and well-understood physiological and pharmacokinetic processes. The system comprises a comprehensive physiology-based model of erythropoiesis and erythrocyte dynamics^29^ that estimates patient-specific key physiological characteristics, such as red blood cell lifespan. For each patient, the system creates a set of personalized models using routine clinical data (sex, height, recent body weights, hemoglobin levels, and ESA doses).^30^ A model predictive controller processes the models' predictions of individual hemoglobin trajectories to provide fully personalized ESA dosing recommendations.^31^ The software is specifically designed to achieve and maintain hemoglobin levels within narrow target ranges using ESA efficiently.

To better understand its clinical performance, we conducted a multicenter, randomized controlled trial comparing our anemia therapy assistance system with a widely used population-based standard-of-care anemia treatment protocol. We tested the hypothesis that personalized ESA dosing improves hemoglobin target attainment.

## Methods

### Study Design

We conducted a multicenter, double-arm, randomized, controlled pilot study in patients on maintenance hemodialysis who were prescribed methoxy polyethylene glycol-epoetin beta (Mircera, Roche, Basel, Switzerland) as their exclusive ESA for anemia management. Participants were recruited from five Fresenius Kidney Care dialysis clinics (Supplemental Table 1), which are part of Fresenius Medical Care North America, a large dialysis organization. In all five clinics, devices to continuously monitor hematocrit concentrations during treatments are used as part of the standard of care (Fresenius Medical Care, Waltham, MA).

The study was reviewed and approved by the Western Institutional Review Board (WIRB Protocol 20192854) and registered at ClinicalTrials.gov (NCT04360902) (ClinicalTrials.gov initial release was on December 19, 2019, and public release was on April 23, 2020. Recruitment was initiated before public release). It was conducted in compliance with Good Clinical Practice and the Declaration of Helsinki. All patients provided written, informed consent.

Once written consent was obtained and all eligibility criteria were verified, participants were randomized in a 1:1 ratio into either the intervention group or the standard-of-care group. Randomization was performed by a trained statistician. A list was created through random sampling without replacement from a set of integers, which was then evenly divided into two groups. Assignment to the study arms was unblinded. In both groups, the target hemoglobin range was 10–11 g/dl. The baseline period consisted of the 180 days before the individual's study start. Each participant remained in the study for a period of 26 weeks. During these 26 weeks, participants in the standard-of-care arm continued to receive standard-of-care anemia management using the clinics' anemia treatment protocol while participants randomized to the intervention arm received individualized anemia management in the form of ESA dose recommendations generated by the anemia therapy assistance software.

### Study Population

Eligible patients were adults (18 years and older) with kidney anemia receiving thrice-weekly in-center hemodialysis. Prospective study participants were required to have received at least two ESA administrations in the 150 days preceding study enrollment. In addition, laboratory hemoglobin and hematocrit data measured by a noninvasive device had to be available during baseline with a combined average frequency of at least two measurements per week. Patients also had to exhibit hemoglobin fluctuations defined as a difference between minimum and maximum hemoglobin levels ≥1.75 g/dl and a weekly hemoglobin rate of change ≥0.1 g/dl for more than 60% of the time during baseline (a detailed description of the assessment of fluctuating hemoglobin levels is provided in the Supplemental Methods).

### Study Procedures

The 26-week study period commenced on the day of the next scheduled decision regarding ESA administration after randomization. ESA dose recommendations, either by the standard-of-care treatment protocol or the software system, were communicated to the clinics by electronic reports and reviewed and processed by the clinics' anemia managers. Iron therapy was provided as per the standard of care in the participating clinics and managed in the same manner in both groups.

### Standard-of-Care Group

Participants in the standard-of-care arm continued to be treated per anemia therapy protocol used in the clinics. This rule-based protocol was the same in all participating clinics. It consists of a set of prewritten guidelines reviewed by the large dialysis organization's medical advisory board. The treatment protocol takes up to the latest three hemoglobin measurements into account. Adjustments to the current ESA dose are suggested on the basis of the most recent hemoglobin level and trend of the hemoglobin curve.

### Intervention Group

For participants in the intervention arm, the therapy assistance software computed individualized ESA dose recommendations fortnightly (for details, see Supplemental Methods). In brief, the software combines a generic physiology-based model, an algorithm to individualize the model on the basis of patient's routine clinical data, and a model predictive controller (Supplemental Figure 1). It is designed to guide and stabilize a patient's hemoglobin level within a predefined target range.

### Study Outcomes

The primary study outcome was the percentage of hemoglobin levels within the target range on a patient level. Secondary outcome measures included the number and percentage of patients with fluctuating hemoglobin levels, mean hemoglobin levels, SD around the participant-specific average hemoglobin level, distance to the hemoglobin target, and ESA utilization.

### Sample Size Estimation

No prior data were available for a formal power analysis. Therefore, we conducted an interim analysis to determine the final sample size. The estimation was based on data collected from 23 patients who had participated in the study for at least 6 weeks (in total 287 laboratory hemoglobin measurements). We used Monte–Carlo simulation in conjunction with mixed-effects logistic regression models to calculate the power with significance level (*α*) of 0.01. We determined that 40 patients in each group would provide >90% power to detect a 20% difference of percentage of hemoglobin values within target.

### Statistical Analysis

Statistical analyses were performed using SAS version 9.4 (SAS Institute, Cary, NC), R i386 3.0.2 and R 4.2.2 (R Foundation for Statistical Computing, Vienna, Austria), and SciPy 1.9.3.

Baseline characteristics were calculated for the full cohort, *i.e*., all randomized participants, whereas the analytical cohort included only patients who contributed at least 30 days of data during the study period. Outcome measures such as hemoglobin control and ESA utilization cannot meaningfully be interpreted in participants who remained in the study for only <30 days. Descriptive statistics were presented as median (25th and 75th percentiles) for continuous variables and counts (percentages) for categorical variables. Continuous variables were averaged through the baseline period per patient and then per group with two exceptions. Monthly ESA doses were determined on 30-day bins and then averaged per patient and then per group. Hospitalization duration (in days) was determined as per event and not as per patient. Wilcoxon rank-sum and chi-square tests were used for group comparisons at baseline. Resampling random pairs from the intervention and standard-of-care groups 20,000 times (with replacement) and calculating a difference using a bootstrapping approach allowed for the calculation of the 95% confidence interval (CI) of the difference between the median values.

Hemoglobin-related outcome measures were evaluated using routine weekly laboratory measurements. For analysis of the primary outcome measure, percentage of measurements within the target range, we conducted univariate analyses using the Wilcoxon rank-sum test and computing the CIs (using the same approach as for the baseline comparison) and constructed a linear mixed-effects logistic regression model to quantify the odds of being in the hemoglobin target in the standard-of-care compared with the intervention group. All secondary outcome measures were analyzed in a univariate fashion and CIs computed using the same approach as for the baseline comparison. The secondary outcome measures distance to the hemoglobin target and ESA utilization were additionally analyzed using a linear mixed-effects regression model to estimate the treatment effect. Missing data were not considered for the analyses. To ascertain robustness of our results, we compared the unadjusted linear mixed-effects models to models adjusted for (*1*) diabetes and (*2*) diabetes and hemoglobin levels at the end of the baseline period. An additional sensitivity analysis was conducted in patients who contributed at least 90 days and in those who completed the study (Supplemental Tables 2 and 3).

## Results

**Figure 1:**
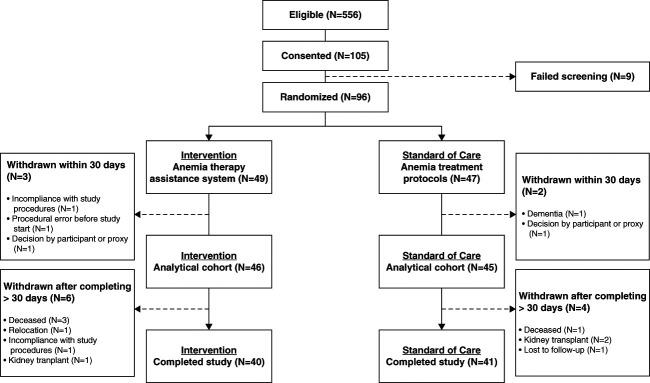
**Summary of the study populations.** Study analysis was performed on the analytical cohort (*i.e*., patients who contributed at least 30 days of data during the study period). The median follow-up period was 26 weeks, with no variability within the IQR, in both groups.

Patients were recruited from five dialysis facilities located in New York (*n*=2), Connecticut (*n*=2), and California (*n*=1) (Supplemental Table 1). Recruitment commenced in January 2020 and ended in July 2021. Of 556 prescreened patients, who were potentially eligible, 96 were randomized (intervention group: *n*=49; standard-of-care group: *n*=47). Enrollment was stopped when a number was reached that was expected to provide a meaningful interpretation of the primary outcome measure on the basis of the power calculation from the interim analysis and adding a safety margin to account for attrition. Ninety-one participants remained at least 30 days in the study and established the analytical cohort (intervention group: *n*=46; standard-of-care group: *n*=45). Eighty-one patients completed the study (completed study cohort; intervention group: *n*=40; standard-of-care group: *n*=41) Figure 1. Reasons for withdrawal of 15 patients were death (*n*=4), kidney transplantation (*n*=3), noncompliance with study protocol (*n*=2), and others (*n*=6). We observed, in total, 47 serious adverse events (intervention group: *n*=20 from 14 participants; standard-of-care group: *n*=27 from 15 participants) and five adverse events (intervention group: *n*=4 from two participants; standard-of-care group: *n*=1 from one participant). All were adjudicated as being unrelated to the study intervention. More details are provided in Supplemental Table 4. Of 44 hospitalizations during the study, five were coronavirus disease 2019–related hospitalizations (intervention group: *n*=3; standard-of-care group: *n*=2). In the intervention group, of 561 ESA dose recommendations generated by the anemia therapy assistance software, only 31 were overridden by clinicians, with six additional deviations due to logistic challenges.

Baseline characteristics were well balanced between groups (Table 1), except for diabetes mellitus (intervention group: *n*=22 [45%], standard-of-care group: *n*=32 [68%]).

**Table 1:** Baseline characteristics of all randomized study participants

Characteristic	Intervention Group, *n*=49	Standard-of-Care Group, *n*=47	Total, *N*=96
Age, yr	62 (55–71)	60 (50–72)	61 (51)
Male	31 (63)	27 (57)	58 (60)
Dialysis vintage, yr	3 (2–7)	3 (1–6)	3 (1–7)
**Dialysis access type**			
Arteriovenous fistula	31 (63)	32 (68)	63 (66)
Arteriovenous graft	8 (16)	8 (17)	16 (17)
Central venous catheter	10 (20)	8 (17)	18 (19)
Body mass index, kg/m^2^	26.9 (21.6–30.5)	26.3 (22.5–30.8)	26.5 (22.1–30.7)
Post-dialysis body weight, kg	74 (59–83)	70 (60–82)	71 (59–83)
Interdialytic weight gain, kg	2.2 (1.7–2.7)	2.2 (1.9–2.8)	2.2 (1.7–2.8)
Hemoglobin at end of baseline, g/dl	11.0 (10–11.8)	10.7 (9.5–12.0)	10.9 (9.8–11.8)
Mean hemoglobin during baseline, g/dl	10.5 (10.1–11)	10.7 (10.3–10.9)	10.6 (10.2–10.9)
Hemoglobin standard deviation, g/dl	0.9 (0.8–1)	0.9 (0.8–1.4)	0.9 (0.8–1.3)
Hemoglobin values in target, %	36 (27–46)	38 (24–49)	37 (26–47)
Hemoglobin distance to target, g/dl	0.4 (0.3–0.6)	0.4 (0.3–0.7)	0.5 (0.3–0.7)
Mean monthly ESA dose, mcg/30 d	93 (55–176)	86 (60–130)	90 (58–143)
Rate of ESA dose administrations, 1/30 d	1.3 (1.0–1.7)	1.3 (1.0–1.5)	1.3 (1.0–1.5)
Iron dose, mg/30 d	208 (75–292)	225 (129–292)	213 (108–292)
Ferritin, ng/ml	805 (484–1067)	893 (499–1265)	839 (490–1168)
Transferrin saturation, %	32 (25–37)	32 (27–41)	32 (26–38)
Serum albumin, g/dl	4.0 (3.8–4.1)	3.9 (3.7–4.1)	3.9 (3.7–4.1)
Neutrophil-to-lymphocyte ratio	3.2 (2.4–4.8)	3.3 (2.7–4.1)	3.2 (2.4–4.8)
spKt/V	1.7 (1.5–1.9)	1.7 (1.6–1.8)	1.7 (1.5–1.9)
Intact PTH, pg/ml	669 (403–928)	507 (339–726)	546 (385–796)
Hospitalizations per 180 d	0 (0–1)	0 (0–1)	0 (0–1)
Hospitalization duration per episode, d	5 (4–7)	3 (2–5)	4 (2–6)
**Diabetes mellitus**			
No	27 (55)	15 (32)	42 (44)
Yes	22 (45)	32 (68)	54 (56)
**Congestive heart failure**			
No	41 (84)	41 (87)	82 (85)
Yes	8 (16)	6 (13)	14 (15)

Data are given as count (percentage) or median (interquartile range) as appropriate. ESA, erythropoiesis-stimulating agent; PTH, parathyroid hormone; spKt/V, single pool Kt/V.

### Primary Outcome Measure

The primary study outcome measure was the percentage of patient-level hemoglobin values within the target range of 10–11 g/dl. The median follow-up period was 26 weeks, with no variability within the interquartile range (IQR), in both groups. Weekly laboratory hemoglobin data were missing in both groups with a similar frequency (*P* = 0.96). During the study period, 4% (IQR, 0–12) of the laboratory hemoglobin data were missing in the intervention group and 4% (IQR, 0–8) in the standard-of-care group; missing data resulted from hospitalizations, skipped visits, and other reasons for missed blood draws.

**Figure 2:**
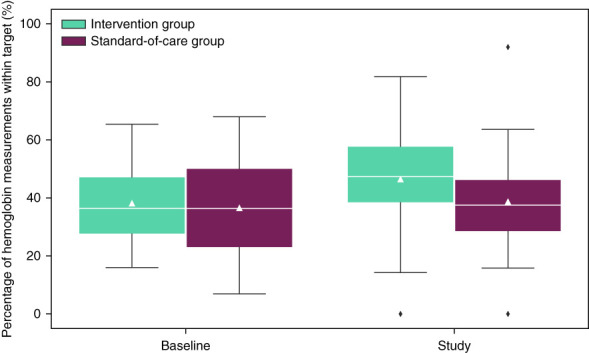
**Primary outcome measure, percentage of hemoglobin within target, improved in the intervention group (analysis cohort).** Triangles indicate the mean and white lines the median values. The boxes show the quartiles of the data and whiskers extend to show the rest of the distribution, except for values outside 1.5 times of the IQR that are depicted as outliers. IQR, interquartile range.

At baseline, the groups were similar with the median percentage of hemoglobin values within target being 36% (IQR, 27–46) in the intervention group and 38% (IQR, 24–49) in the standard-of-care group. During the study, target attainment in the intervention group improved to 47% (IQR, 39–58), whereas the standard-of-care group remained the same (38%; IQR, 29–46) as compared with baseline (Figure 2). The resulting median difference of 10 percentage points (95% CI, 3 to 16) between the two groups was nominally statistically significant (*P* = 0.008) (Table 2). These findings translate into a relative increase of percentage of hemoglobin values within target of approximately 25%. The odds ratio of achieving the hemoglobin target in the standard-of-care group compared with the intervention group was 0.68 (95% CI, 0.51 to 0.92) (*P* = 0.01); models adjusted for diabetes 0.70 (95% CI, 0.52 to 0.94) (*P* = 0.02) and for diabetes and hemoglobin levels at baseline 0.69 (95% CI, 0.51 to 0.93) (*P* = 0.01) showed similar results.

**Table 2:** Primary and secondary outcome measures (analytical cohort)

Characteristic	Intervention Group, *n*=46	Standard-of-Care Group, *n*=45	Median Difference (95% CI)	*P* Value
**Primary outcome measure**				
Hemoglobin values in target, %	47 (39–58)	38 (29–46)	10 (3 to 16)	0.008
**Secondary outcome measures**				
Patients with fluctuating hemoglobin levels, % (intervention group: *n*=44, standard-of-care group: *n*=44)	45	82	−37 (−54 to −17)	<0.001
Mean hemoglobin, g/dl	10.4 (10.2–10.7)	10.7 (10.4–11.1)	−0.3 (−0.4 to −0.008)	0.03
Hemoglobin standard deviation, g/dl	0.7 (0.6–0.9)	0.9 (0.7–1.1)	−0.2 (−0.3 to −0.04)	<0.001
Hemoglobin distance to target, g/dl	0.3 (0.2–0.4)	0.4 (0.3–0.7)	−0.2 (−0.3 to −0.05)	<0.001
Mean ESA dose, mcg/30 d	69 (47–114)	91 (60–148)	−23 (−67 to 5)	0.06
Mean ESA dose, mcg/30 d per kilogram	1.0 (0.6–1.5)	1.5 (0.9–2.2)	−0.4 (−0.8 to 0.1)	0.02

Data are given as median (interquartile range). Bootstrapping was used to calculate the median difference and the 95% confidence interval of the difference. Group comparisons were performed by Wilcoxon rank-sum and chi-square tests as appropriate. CI, confidence interval; ESA, erythropoiesis-stimulating agent.

### Secondary Outcome Measures

**Figure 3:**
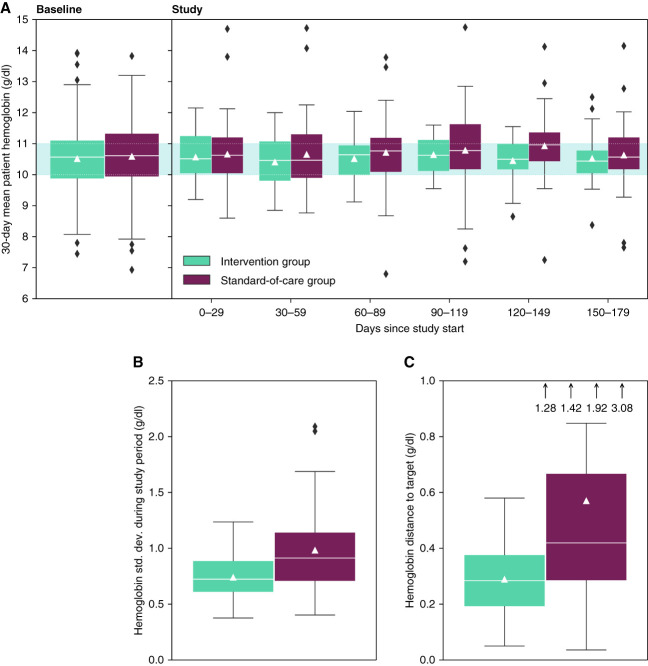
**Secondary outcome measures related to hemoglobin variability improved in the intervention group (completed study population).** Triangles indicate the mean and white lines the median values. The boxes show the quartiles of the data and whiskers extend to show the rest of the distribution, except for values outside 1.5 times of the IQR that are depicted as outliers. (A) Progression of the mean monthly hemoglobin levels per patient throughout the study. The blue shaded area indicates the hemoglobin target of 10–11 g/dl. (B and C) Hemoglobin SD and hemoglobin distance to the target for each patient during the study period. Four outliers in (C) are shown by arrows and numerical values.

The stability of hemoglobin levels, quantified as the percentage of patients experiencing fluctuating hemoglobin levels, significantly improved in the intervention group with 45% versus 82% in the standard-of-care group (*P* < 0.001). Both inter- and intraindividual hemoglobin precision increased in the intervention arm (Table 2). Mean monthly hemoglobin levels per patient progressively narrowed and centered in the hemoglobin target range in the intervention group (Figure 3). Intrapatient hemoglobin variability, defined as the SD around the mean hemoglobin level, improved in the intervention group compared with the standard-of-care group (median treatment difference: −0.2 g/dl; 95% CI, −0.3 to −0.04; *P* < 0.001). The intervention arm showed greater precision in target attainment with a significant reduction of 0.3 g/dl (95% CI, −0.4 to −0.1) in the distance to the hemoglobin target in unadjusted (*P* = 0.003) and adjusted (*P* = 0.002) models.

**Figure 4:**
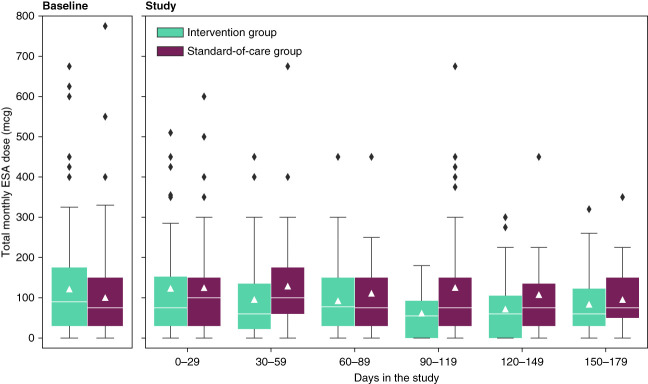
**Secondary outcome measure: Monthly ESA doses were consistently lower in the intervention group compared with the standard-of-care group (completed study population).** Triangles indicate the mean and white lines the median values. The boxes show the quartiles of the data and whiskers extend to show the rest of the distribution, except for values outside 1.5 times of the IQR that are depicted as outliers. ESA, erythropoiesis-stimulating agent.

We observed a clinically significant trend toward lower ESA utilization in the intervention group compared with the standard-of-care group. The difference in ESA utilization did not reach statistical significance, except when normalized by weight (Table 2). The mean observed treatment effect in the unadjusted model was −29 mcg/30 days (95% CI, −60 to 2; *P* = 0.07); in the model adjusted for diabetes, it was −27 mcg/30 days (95% CI, −58 to 5; *P* = 0.10); and in the model adjusted for diabetes and hemoglobin levels at baseline, it was −22 mcg/30 days (95% CI, −50 to 6; *P* = 0.09). Throughout the study period, monthly ESA doses were consistently lower in the intervention arm. The IQRs narrowed with the use of the personalized recommendation tool compared with baseline and compared with the standard-of-care arm, respectively (Figure 4).

### Other Outcomes

ESA therapy–related parameters, such as rate of ESA administration, iron supplementation, and ferritin concentration, were similar in both groups during the study period (Table 3). The transferrin saturation showed a nonsignificant trend of being slightly lower in the intervention arm compared with the standard-of-care arm (median difference, −4.0%; 95% CI, −11.0 to 2.0; *P* = 0.12).

**Table 3:** Other outcomes (analytical cohort)

Characteristic	Intervention Group, *n*=46	Standard-of-Care Group, *n*=45	Median Difference (95% CI)	*P* Value
Rate of ESA administration, per 30 d	1.4 (0.8–1.6)	1.3 (1–1.6)	0.03 (−0.3 to 0.3)	0.91
Iron dose, mg/30 d	163 (58–229)	142 (75–225)	25 (−50 to 83)	0.89
Ferritin concentration, ng/ml	849 (567–1053)	765 (608–1249)	70 (−277 to 212)	0.48
Transferrin saturation, % (intervention group: *n*=41; standard-of-care group: *n*=43)	29 (25–39)	33 (27–45)	−4 (−11 to 2)	0.12

Data are presented as median (interquartile range). Bootstrapping was used to calculate the median difference and the 95% confidence interval of the difference. Group comparisons were performed by Wilcoxon rank-sum test. CI, confidence interval; ESA, erythropoiesis-stimulating agent.

## Discussion

The results of our randomized clinical pilot trial show that the personalized ESA recommendations by a physiology-based mathematical model of erythropoiesis combined with a model predictive controller significantly increased the rate of target hemoglobin attainment compared with the standard of care. In addition, both inter- and intrapatient hemoglobin variability significantly improved in the intervention arm, thus increasing the attainment of hemoglobin values within the target range and reducing the overall distance to the hemoglobin target.

We also observed a trend toward lower ESA utilization in the intervention group compared with the standard-of-care group, which was clinically relevant but only statistically significant when normalized by patient weight. A tighter hemoglobin control and centering of mean hemoglobin levels closer to the midpoint of the target range are possible explanation for the reduced ESA utilization in the intervention group.

Our trial focused on patients on hemodialysis who previously had received ESA therapy and exhibited fluctuating hemoglobin levels. At the time the study commenced (January 2020), fluctuating hemoglobin levels were observed in 48%–70% of the patients treated for anemia with methoxy polyethylene glycol-epoetin beta in the five participating clinics. The total percentage of patients exhibiting hemoglobin fluctuations across the five clinics was 59%.

Over the past years, several studies were performed to improve the attainment of predefined hemoglobin targets.^24–26,28,32–36^ These studies used several approaches, including modeling of ESA pharmacokinetics and machine learning. However, the anemia therapy assistance system developed by us and evaluated rigorously in this randomized controlled trial differs from earlier approaches with respect to several innovations. First, we developed and used in the clinical pilot trial a novel, general, deterministic, physiology-based mathematical model that describes the physiology of erythropoiesis and red blood cell biology.^29^ Second, this model was combined with a general ESA pharmacokinetic and pharmacodynamic model. Third, using a specific patient's data, these general models were then personalized, creating a patient's digital twin, a.k.a. “patient avatar.”^30^ Fourth, model predictive control theory^31^ was then used to compute optimal ESA doses by simulating the avatar's hemoglobin response to ESA. To the best of our knowledge, the novelty and originality of our approach is the seamless integration of personalized erythropoiesis and ESA pharmacokinetic/pharmacodynamic models with model predictive control to attain a predefined hemoglobin target. As a by-product, the recurrent patient-specific model adaptations that are part of the anemia therapy assistance software, yield repeated estimates of physiological parameters, such as red blood cell lifespan or ESA *t*_1/2_. The exploration of the clinical utility of these digital biomarkers merits further investigation.

Studies assessing these other decision-making systems are difficult to compare with our study for several reasons. First, the patient populations are different. Second, regarding anemia management, the hemoglobin target ranges (both midpoint and window size), hemoglobin measurement frequencies, ESA type, and anemia treatment schemes in the respective control groups differ. Finally, and most importantly, we conducted a randomized controlled trial and not a comparison of parameters between pre- and post-implementation periods. These factors have major effects on both absolute and relative improvements in hemoglobin target attainment and ESA dose reduction. An excellent review of computerized systems evaluated in clinical studies was provided by Brier *et al.* in 2018.^37^

Strengths of our study are its randomized, multicenter design; a balanced rate of study discontinuation in both arms; and the use of a narrow hemoglobin target of 10–11 g/dl, complies with the US Food and Drug Administration black box warning for ESAs. This target range is narrower than any other guideline or consensus-based recommendation and more challenging to achieve and maintain.

Some limitations of our study warrant recognition. First, the use of methoxy polyethylene glycol-epoetin beta as the exclusive ESA for anemia management and the fact that all study participants were recruited from US Fresenius Kidney Care dialysis clinics may limit the generalizability of the results to other dialysis providers and other ESAs. Second, our enrollment focused on patients with fluctuating hemoglobin levels who had received anemia therapy for at least 6 months. Third, selection of clinics was limited to those that use devices to noninvasively monitor hematocrit concentrations during treatments as part of their standard of care. Fourth, despite randomization, the frequency of diabetes mellitus was significantly higher in the standard-of-care arm. Although not a limitation *per se*, we want to point out that the study was not designed and powered to compare clinical outcomes (*e.g*., cardiovascular morbidity) between the two groups. Furthermore, the system was tested in clinics where weekly laboratory hemoglobin measurements and frequent use of devices to noninvasively monitor hematocrit concentration during treatments was part of the standard of care. This might not be true for other clinics or providers and could lead to additional costs if adopted. However, these costs might be offset by potential ESA savings observed in this study. Finally, the study investigators were not blinded to the treatment group allocation after randomization.

In summary, in comparison with standard-of-care anemia management, our study has shown superior performance of anemia management built on physiology-based mathematical models combined with a model predictive controller. The implementation of this approach in clinical practice merits further consideration and exploration.

## Supplementary Material

## Data Availability

Partial restrictions to the data and/or materials apply. Aggregated patient-level summary data underlying this article are available upon request to the authors (this includes the patient-level information on counts, means, standard deviations, 25th, 50th, and 75th percentiles). Access to individual data on a point measure level is restricted. The Renal Research Institute will make these data available upon reasonable request and subject to institutional approval. Interested parties will also need to sign a data use agreement to access patient individual data.

## Supplemental Material

Supplemental Methods

• Inclusion and exclusion criteria.

• Assessment of fluctuating hemoglobin levels.

• Anemia therapy assistance software.

Supplemental Analysis

• Additional analysis of the primary and secondary outcomes.

Supplemental Table 1. Clinic names and locations of clinics that participated in the randomized controlled trial.

Supplemental Table 2. Primary and secondary outcomes (patients who remained in the study for ≥90 days).

Supplemental Table 3. Primary and secondary outcomes (patients who completed the study).

Supplemental Table 4. List of adverse and serious adverse events during the study.

Supplemental Figure 1. Schematic of the anemia therapy assistance system.
