# Supplementary material for: Effects of Individualized Anemia Therapy on Hemoglobin Stability: A Randomized Controlled Pilot Trial in Patients on Hemodialysis
Source: Clin J Am Soc Nephrol. 2024 Jun 11;19(9):1138–47. doi: 10.2215/CJN.0000000000000488 (PMC11390026; doi:10.2215/CJN.0000000000000488)
Supplement: Supplementary file 1 [file cjasn-19-1138-s001.pdf]

ASN American Society of Nephrology | JASN Journal of the American Society of Nephrology | KIDNEY360 Accessing Our World From Every Angle | CJASN Clinical Journal of the American Society of Nephrology

ASN Journal Disclosure Form

As per ASN journal policy, I have disclosed any financial relationships or commitments I have held in the past 36 months as included below. I have listed my Current Employer below to indicate there is a relationship requiring disclosure. If no relationship exists, my Current Employer is not listed.

S. Casper reports the following:
Employer: Fresenius Medical Care Deutschland GmbH; and Patents or Royalties: Fresenius Medical Care.

I understand that the information above will be published within the journal article, if accepted, and that failure to comply and/or to accurately and completely report the potential financial conflicts of interest could lead to the following: 1) Prior to publication, article rejection, or 2) Post-publication, sanctions ranging from, but not limited to, issuing a correction, reporting the inaccurate information to the authors' institution, banning authors from submitting work to ASN journals for varying lengths of time, and/or retraction of the published work.

Name: Sabrina Casper
Manuscript ID: CJASN-2023-001692R5
Manuscript Title: Effects of individualized anemia therapy on hemoglobin stability: a randomized controlled pilot trial in hemodialysis patients.
Date of Completion: June 3, 2024
Disclosure Updated Date: April 9, 2024

ASN Journal Disclosure Form

As per ASN journal policy, I have disclosed any financial relationships or commitments I have held in the past 36 months as included below. I have listed my Current Employer below to indicate there is a relationship requiring disclosure. If no relationship exists, my Current Employer is not listed.

A. Cherif reports the following:
Employer: Merck & Co., Inc; Renal Research Institute; Ownership Interest: I owe stocks in Merck & Co., Inc.; and Patents or Royalties: Renal Research Institute.

I understand that the information above will be published within the journal article, if accepted, and that failure to comply and/or to accurately and completely report the potential financial conflicts of interest could lead to the following: 1) Prior to publication, article rejection, or 2) Post-publication, sanctions ranging from, but not limited to, issuing a correction, reporting the inaccurate information to the authors' institution, banning authors from submitting work to ASN journals for varying lengths of time, and/or retraction of the published work.

Name: Alhaji Cherif
Manuscript ID: CJASN-2023-001692R5
Manuscript Title: Effects of individualized anemia therapy on hemoglobin stability: a randomized controlled pilot trial in hemodialysis patients.
Date of Completion: May 31, 2024
Disclosure Updated Date: May 31, 2024

ASN Journal Disclosure Form

As per ASN journal policy, I have disclosed any financial relationship or commitment held by myself and/or my spouse/partner in the past 36 months as included below. I have listed my Current Employer below to indicate there is a relationship requiring disclosure. If no relationship exists, my Current Employer is not listed.

D. Fuertinger reports the following:
Employer: Fresenius Medical Care D-GmbH; Research Funding: Fresenius Medical Care; and Patents or Royalties: Multiple patents in the kidney space in the area of treatment of anemia, bone mineral disease, fluid management, and conduct of simulated clinical studies.

I understand that the information above will be published within the journal article, if accepted, and that failure to comply and/or to accurately and completely report the potential financial conflicts of interest could lead to the following: 1) Prior to publication, article rejection, or 2) Post-publication, sanctions ranging from, but not limited to, issuing a correction, reporting the inaccurate information to the authors' institution, banning authors from submitting work to ASN journals for varying lengths of time, and/or retraction of the published work.

Name: Doris H. Fuertinger
Manuscript ID: CJASN-2023-001692R1
Manuscript Title: Effects of individualized anemia therapy on hemoglobin stability: a randomized controlled pilot trial in hemodialysis patients.
Date of Completion: January 25, 2024
Disclosure Updated Date: January 25, 2024

ASN American Society of Nephrology
JASN Journal of the American Society of Nephrology
KIDNEY360 Accessing Our World From Every Angle
CJASN Clinical Journal of the American Society of Nephrology

ASN Journal Disclosure Form

As per ASN journal policy, I have disclosed any financial relationships or commitments I have held in the past 36 months as included below. I have listed my Current Employer below to indicate there is a relationship requiring disclosure. If no relationship exists, my Current Employer is not listed.

K. Ho reports the following:
Employer: Fresenius Medical Care North America; Advisory or Leadership Role: Fresenius Medical Care North America; and Other Interests or Relationships: American Society of Nephrology; National Kidney Foundation.

I understand that the information above will be published within the journal article, if accepted, and that failure to comply and/or to accurately and completely report the potential financial conflicts of interest could lead to the following: 1) Prior to publication, article rejection, or 2) Post-publication, sanctions ranging from, but not limited to, issuing a correction, reporting the inaccurate information to the authors' institution, banning authors from submitting work to ASN journals for varying lengths of time, and/or retraction of the published work.

Name: Kevin Ho
Manuscript ID: CJASN-2023-001692R3
Manuscript Title: Effects of individualized anemia therapy on hemoglobin stability: a randomized controlled pilot trial in hemodialysis patients
Date of Completion: May 10, 2024
Disclosure Updated Date: April 2, 2024

ASN American Society of Nephrology | JASN Journal of the American Society of Nephrology | KIDNEY360 Accessing Our World From Every Angle | CJASN Clinical Journal of the American Society of Nephrology

ASN Journal Disclosure Form

As per ASN journal policy, I have disclosed any financial relationships or commitments I have held in the past 36 months as included below. I have listed my Current Employer below to indicate there is a relationship requiring disclosure. If no relationship exists, my Current Employer is not listed.

D. Joerg reports the following:
Employer: Fresenius Medical Care Germany; Ownership Interest: A full list of individual stocks held during the last 24 months exceeds the character limit and is available upon request.; Research Funding: Fresenius Medical Care; and Patents or Royalties: "A system for determining the magnitude of the ultrafiltration volume expected in a peritoneal dialysis treatment" (WO 2023/126465 A1); "SYSTEM AND METHOD FOR ADJUSTING HYPOXIA-INDUCIBLE FACTOR STABILIZER TREATMENT BASED ON ANEMIA MODELING" (US Patent App. 17/960,305); "SYSTEM AND METHOD FOR ATTAINING DESIRED OXYGEN DOSING BASED ON ERYTHROPOIESIS MODELING" (US Patent App. 17/960,560); "TECHNIQUES FOR INCREASING RED BLOOD CELL COUNT" (US Patent App. 18/032,931).

I understand that the information above will be published within the journal article, if accepted, and that failure to comply and/or to accurately and completely report the potential financial conflicts of interest could lead to the following: 1) Prior to publication, article rejection, or 2) Post-publication, sanctions ranging from, but not limited to, issuing a correction, reporting the inaccurate information to the authors' institution, banning authors from submitting work to ASN journals for varying lengths of time, and/or retraction of the published work.

Name: David J. Joerg
Manuscript ID: #CJASN-2023-001692R5
Manuscript Title: Effects of individualized anemia therapy on hemoglobin stability: a randomized controlled pilot trial in hemodialysis patients
Date of Completion: May 28, 2024
Disclosure Updated Date: May 8, 2024

ASN Journal Disclosure Form

As per ASN journal policy, I have disclosed any financial relationships or commitments I have held in the past 36 months as included below. I have listed my Current Employer below to indicate there is a relationship requiring disclosure. If no relationship exists, my Current Employer is not listed.

P. Kotanko reports the following:
Employer: Renal Research Institute; Ownership Interest: Fresenius Medical Care; Research Funding: Fresenius Medical Care; NIH; KidneyX; PCORI; Patents or Royalties: Multiple patents in the kidney space; and Advisory or Leadership Role: Editorial Board of Blood Purification; Editorial Board of Kidney and Blood Pressure Research; Editorial Board of Frontiers in Nephrology.

I understand that the information above will be published within the journal article, if accepted, and that failure to comply and/or to accurately and completely report the potential financial conflicts of interest could lead to the following: 1) Prior to publication, article rejection, or 2) Post-publication, sanctions ranging from, but not limited to, issuing a correction, reporting the inaccurate information to the authors' institution, banning authors from submitting work to ASN journals for varying lengths of time, and/or retraction of the published work.

Name: Peter Kotanko
Manuscript ID: CJASN-2023-001692R5
Manuscript Title: Effects of individualized anemia therapy on hemoglobin stability: a randomized controlled pilot trial in hemodialysis patients.
Date of Completion: May 31, 2024
Disclosure Updated Date: May 6, 2024

ASN American Society of Nephrology JASN Journal of the American Society of Nephrology KIDNEY360 Accessing Our World From Every Angle CJASN Clinical Journal of the American Society of Nephrology

ASN Journal Disclosure Form

As per ASN journal policy, I have disclosed any financial relationships or commitments I have held in the past 36 months as included below. I have listed my Current Employer below to indicate there is a relationship requiring disclosure. If no relationship exists, my Current Employer is not listed.

A. Mermelstein reports the following:
Employer: Renal Research Institute; and Research Funding: Vifor Fresenius Medical Care Renal Pharma.

I understand that the information above will be published within the journal article, if accepted, and that failure to comply and/or to accurately and completely report the potential financial conflicts of interest could lead to the following: 1) Prior to publication, article rejection, or 2) Post-publication, sanctions ranging from, but not limited to, issuing a correction, reporting the inaccurate information to the authors' institution, banning authors from submitting work to ASN journals for varying lengths of time, and/or retraction of the published work.

Name: Ariella E. Mermelstein
Manuscript ID: CJASN-2023-001692R5
Manuscript Title: Effects of individualized anemia therapy on hemoglobin stability: a randomized controlled pilot trial in hemodialysis patients.
Date of Completion: May 28, 2024
Disclosure Updated Date: May 28, 2024

ASN American Society of Nephrology CJASN Kidney360 JASN

ASN Journal Disclosure Form

As per ASN journal policy, I have disclosed any financial relationship or commitment held by myself and/or my spouse/partner in the past 36 months as included below. I have listed my Current Employer below to indicate there is a relationship requiring disclosure. If no relationship exists, my Current Employer is not listed.

J. Raimann reports the following:
Employer: Renal Research Institute, a wholly owned subsidiary of Fresenius Medical Care; Ownership Interest: owning shares of stock in Fresenius Medical Care; and Other Interests or Relationships: Member of the Board of Directors "Easy Water for Everyone" (501c3).

I understand that the information above will be published within the journal article, if accepted, and that failure to comply and/or to accurately and completely report the potential financial conflicts of interest could lead to the following: 1) Prior to publication, article rejection, or 2) Post-publication, sanctions ranging from, but not limited to, issuing a correction, reporting the inaccurate information to the authors' institution, banning authors from submitting work to ASN journals for varying lengths of time, and/or retraction of the published work.

Name: Jochen G. Raimann
Manuscript ID: CJASN-2023-001692
Manuscript Title: Effects of individualized anemia therapy on hemoglobin stability and ESA utilization in hemodialysis patients
Date of Completion: December 20, 2023
Disclosure Updated Date: December 20, 2023

ASN American Society of Nephrology
JASN Journal of the American Society of Nephrology
KIDNEY360 Accessing Our World From Every Angle
CJASN Clinical Journal of the American Society of Nephrology

ASN Journal Disclosure Form

As per ASN journal policy, I have disclosed any financial relationships or commitments I have held in the past 36 months as included below. I have listed my Current Employer below to indicate there is a relationship requiring disclosure. If no relationship exists, my Current Employer is not listed.

L. Rivera Fuentes reports the following:
Employer: Renal Research Institute, LLC; Applied Therapeutics, Inc; Ownership Interest: Applied Therapeutics, Inc; Research Funding: Renal Research Institute, LLC;; Applied Therapeutics, Inc; and Honoraria: Renal Research Institute, LLC; Applied Therapeutics, Inc.

I understand that the information above will be published within the journal article, if accepted, and that failure to comply and/or to accurately and completely report the potential financial conflicts of interest could lead to the following: 1) Prior to publication, article rejection, or 2) Post-publication, sanctions ranging from, but not limited to, issuing a correction, reporting the inaccurate information to the authors' institution, banning authors from submitting work to ASN journals for varying lengths of time, and/or retraction of the published work.

Name: Lemuel Rivera Fuentes
Manuscript ID: CJASN-2023-001692R5
Manuscript Title: Effects of individualized anemia therapy on hemoglobin stability: a randomized controlled pilot trial in hemodialysis patients
Date of Completion: May 28, 2024
Disclosure Updated Date: May 9, 2024

ASN American Society of Nephrology JASN Journal of the American Society of Nephrology KIDNEY360 Accessing Our World From Every Angle CJASN Clinical Journal of the American Society of Nephrology

ASN Journal Disclosure Form

As per ASN journal policy, I have disclosed any financial relationships or commitments I have held in the past 36 months as included below. I have listed my Current Employer below to indicate there is a relationship requiring disclosure. If no relationship exists, my Current Employer is not listed.

S. Thijssen reports the following:
Employer: Renal Research Institute (affiliated with Fresenius Medical Care North America); Research Funding: Fresenius Medical Care; Patents or Royalties: Fresenius Medical Care Holdings, Inc.; Fresenius Medical Care has patents and patent applications in various stages of the patent prosecution process. I am an inventor on some of these patents and/or patent applications but have assigned my ownership interest to Fresenius Medical Care. I have received and/or may be entitled to a nominal patent award per Fresenius Medical Care North America policy, but I receive no royalties and have no ownership interest in any of this intellectual property.; and Other Interests or Relationships: I hold performance shares (virtual shares) in Fresenius Medical Care.

I understand that the information above will be published within the journal article, if accepted, and that failure to comply and/or to accurately and completely report the potential financial conflicts of interest could lead to the following: 1) Prior to publication, article rejection, or 2) Post-publication, sanctions ranging from, but not limited to, issuing a correction, reporting the inaccurate information to the authors' institution, banning authors from submitting work to ASN journals for varying lengths of time, and/or retraction of the published work.

Name: Stephan Thijssen
Manuscript ID: CJASN-2023-001692R5
Manuscript Title: Effects of individualized anemia therapy on hemoglobin stability: a randomized controlled pilot trial in hemodialysis patients
Date of Completion: May 31, 2024
Disclosure Updated Date: May 13, 2024

ASN American Society of Nephrology | JASN Journal of the American Society of Nephrology | KIDNEY360 Accessing Our World From Every Angle | CJASN Clinical Journal of the American Society of Nephrology

ASN Journal Disclosure Form

As per ASN journal policy, I have disclosed any financial relationships or commitments I have held in the past 36 months as included below. I have listed my Current Employer below to indicate there is a relationship requiring disclosure. If no relationship exists, my Current Employer is not listed.

L. Tisdale reports the following:
Employer: Renal Research Institute

I understand that the information above will be published within the journal article, if accepted, and that failure to comply and/or to accurately and completely report the potential financial conflicts of interest could lead to the following: 1) Prior to publication, article rejection, or 2) Post-publication, sanctions ranging from, but not limited to, issuing a correction, reporting the inaccurate information to the authors' institution, banning authors from submitting work to ASN journals for varying lengths of time, and/or retraction of the published work.

Name: Lela Tisdale
Manuscript ID: CJASN-2023-001692R5
Manuscript Title: Effects of individualized anemia therapy on hemoglobin stability: a randomized controlled pilot trial in hemodialysis patients.
Date of Completion: June 4, 2024
Disclosure Updated Date: June 4, 2024

ASN American Society of Nephrology CJASN Kidney360 JASN

ASN Journal Disclosure Form

As per ASN journal policy, I have disclosed any financial relationship or commitment held by myself and/or my spouse/partner in the past 36 months as included below. I have listed my Current Employer below to indicate there is a relationship requiring disclosure. If no relationship exists, my Current Employer is not listed.

L. Wang reports the following:
Employer: Renal Research Institute

I understand that the information above will be published within the journal article, if accepted, and that failure to comply and/or to accurately and completely report the potential financial conflicts of interest could lead to the following: 1) Prior to publication, article rejection, or 2) Post-publication, sanctions ranging from, but not limited to, issuing a correction, reporting the inaccurate information to the authors' institution, banning authors from submitting work to ASN journals for varying lengths of time, and/or retraction of the published work.

Name: Lin-Chun Wang
Manuscript ID: CJASN-2023-001692
Manuscript Title: Effects of individualized anemia therapy on hemoglobin stability and ESA utilization in hemodialysis patients
Date of Completion: December 20, 2023
Disclosure Updated Date: December 20, 2023

ASN American Society of Nephrology | JASN Journal of the American Society of Nephrology | KIDNEY360 Accessing Our World From Every Angle | CJASN Clinical Journal of the American Society of Nephrology

ASN Journal Disclosure Form

As per ASN journal policy, I have disclosed any financial relationships or commitments I have held in the past 36 months as included below. I have listed my Current Employer below to indicate there is a relationship requiring disclosure. If no relationship exists, my Current Employer is not listed.

X. Ye reports the following:
Employer: Renal Research Institute

I understand that the information above will be published within the journal article, if accepted, and that failure to comply and/or to accurately and completely report the potential financial conflicts of interest could lead to the following: 1) Prior to publication, article rejection, or 2) Post-publication, sanctions ranging from, but not limited to, issuing a correction, reporting the inaccurate information to the authors' institution, banning authors from submitting work to ASN journals for varying lengths of time, and/or retraction of the published work.

Name: Xiaoling Ye
Manuscript ID: CJASN-2023-001692R4
Manuscript Title: Effects of individualized anemia therapy on hemoglobin stability: a randomized controlled pilot trial in hemodialysis patients
Date of Completion: May 28, 2024
Disclosure Updated Date: May 10, 2024

ASN American Society of Nephrology
JASN Journal of the American Society of Nephrology
KIDNEY360 Accessing Our World From Every Angle
CJASN Clinical Journal of the American Society of Nephrology

ASN Journal Disclosure Form

As per ASN journal policy, I have disclosed any financial relationships or commitments I have held in the past 36 months as included below. I have listed my Current Employer below to indicate there is a relationship requiring disclosure. If no relationship exists, my Current Employer is not listed.

H. Zhang reports the following:
Employer: Renal Research Institute; and Patents or Royalties: Renal Research Institute.

I understand that the information above will be published within the journal article, if accepted, and that failure to comply and/or to accurately and completely report the potential financial conflicts of interest could lead to the following: 1) Prior to publication, article rejection, or 2) Post-publication, sanctions ranging from, but not limited to, issuing a correction, reporting the inaccurate information to the authors' institution, banning authors from submitting work to ASN journals for varying lengths of time, and/or retraction of the published work.

Name: Hanjie Zhang
Manuscript ID: CJASN-2023-001692R5
Manuscript Title: Effects of individualized anemia therapy on hemoglobin stability: a randomized controlled pilot trial in hemodialysis patients.
Date of Completion: May 28, 2024
Disclosure Updated Date: May 10, 2024
