# Supplementary material for: Effects of Individualized Anemia Therapy on Hemoglobin Stability: A Randomized Controlled Pilot Trial in Patients on Hemodialysis
Source: Clin J Am Soc Nephrol. 2024 Jun 11;19(9):1138–47. doi: 10.2215/CJN.0000000000000488 (PMC11390026; doi:10.2215/CJN.0000000000000488)
Supplement: Supplementary file 2 [file cjasn-19-1138-s002.pdf]

Supplemental Material

TABLE OF CONTENTS

**Title**	**Page**
Supplemental Methods	1
Inclusion and exclusion criteria	1
Assessment of fluctuating hemoglobin levels	3
Anemia therapy assistance software	4
Supplemental analysis	6
Additional analysis of the primary and secondary outcomes	6
Supplemental Table 1. Clinic names and locations of clinics that participated in the randomized controlled trial.	5
Supplemental Table 2. Primary and secondary outcomes (patients that remained in the study ≥90 days).	6
Supplemental Table 3. Primary and secondary outcomes (patients that completed the study).	7
Supplemental Table 1 Adverse and serious adverse events	7
Supplemental Figure 1 Schematic of the anemia therapy assistance system	8
References	8

Supplemental Materials and Methods

Inclusion and exclusion criteria

Inclusion Criteria

The following inclusion criteria must be met for each subject:

- Age ≥18 years
- Ability to give written informed consent to the study.

- End-stage renal disease treated with thrice-weekly hemodialysis for at least 180 days preceding enrollment.
- Receiving non-invasive monitoring of intradialytic hematocrit concentration, with available hematocrit data going back at least 180 days from the date of enrollment.
- Laboratory hemoglobin data going back at least 180 days from the date of enrollment.
- On average, hemoglobin values available from at least two per week (from either source, non-invasive hematocrit monitoring or laboratory hemoglobin) during the past 180 days preceding enrollment.
- Renal anemia treated with intravenous ESA, with at least two ESA dose administrations during the 150 days preceding enrollment.
- On an active ESA anemia management algorithm order during the 180 days preceding enrollment.
- Patient received exclusively on Mircera® (no other ESAs) during the 180 days preceding enrollment.
- Pattern of fluctuating hemoglobin levels as defined above during the 180 days preceding enrollment.

Exclusion Criteria

Potential study participants will not be included in the study under the following circumstances:

- Having received the maximum ESA® dose (225 µg every other week) consistently throughout the 90 days preceding enrollment.
- Hospitalization for more than 10 days during the 30 days preceding enrollment.
- Severe iron deficiency (TSAT <20%, ferritin <100 ng/mL) in the most recent routine blood work prior to enrollment.
- Any known cause of ESA resistance other than iron deficiency and inflammatory states (e.g. hematologic malignancies, hypersplenism, antibody-mediated pure red cell aplasia).
- Simultaneous participation in another clinical study that may impact anemia management or the outcomes of this trial.
- Inability to communicate in English or Spanish.

Assessment of fluctuating hemoglobin levels

A patient's hemoglobin (hemoglobin) time series is classified as "fluctuating" within a specified time window if the following two criteria are simultaneously fulfilled:

1. The hemoglobin difference between the minimum and the maximum of the time series is larger than 1.75 g/dL.
2. The fraction of time that the weekly hemoglobin rate of change exceeds 0.1 g/dL/week is larger than 60%.

Both criteria are evaluated on a smoothed hemoglobin time series.

Time series smoothing

- The basis for the evaluation of the above criteria is a combined laboratory hemoglobin and hematocrit time series, where the hematocrit measurements are derived from from non-invasive continuous blood monitoring during the treatment. Laboratory hemoglobin measurements are used where available and hematocrit measurements otherwise. Hemoglobin values are computed from hematocrit (Hct) measurements as follows:
    - Both Hct (in units of %) at the start and the end of the dialysis treatment are lowpass-filtered, removing all data points that lie further than 5 percentage points from a moving average computed in a window +/- 10 days around the respective data point.
    - Laboratory hemoglobin (in units of g/dl) is lowpass-filtered, removing all data points that lie further than 5/3 g/dl from a moving average computed in a window +/- 15 days around the respective data point.
    - Hct values at the end of treatment are ignored if there was an unexpected treatment end at the day of the measurement.
    - A time series containing days and measurements where hemoglobin from all three sources (laboratory and continuous monitoring measurements) are present is created.
    - The following steps are carried out for a moving time window of 150 days length starting from the beginning of this time series:
        - Both Hct values (in units of %) are divided by 3 %/(g/dl) to convert to hemoglobin (in units of g/dl), henceforth called "pre- and post-treatment hemoglobin".

        - The mean offset between the pre-treatment hemoglobin and the laboratory hemoglobin values are computed and subtracted from the pre- and post-treatment hemoglobin to align its mean with laboratory hemoglobin.
    - A single interpolation factor between the pre- and post-treatment hemoglobin is computed such that the average deviation between the resulting interpolated hemoglobin and lab hemoglobin is minimized. Both the resulting pre- and post-treatment hemoglobin and laboratory hemoglobin are combined to a single time series using lab hemoglobin values on days where it is available and pre- and post-treatment hemoglobin values otherwise.
- The resulting time series must contain at least 15 data points for the computation of the fluctuating status.
- A spline interpolation of the time series is generated using Python via the SciPy function *scipy.interpolate.splrep* with smoothing parameter *s* = 0.18 * (number of hemoglobin data points). Both the resulting hemoglobin spline and its first derivative are evaluated on a time grid with spacing 0.1 days.

Criteria testing

- Criterion #1 is fulfilled if the difference between the maximum and the minimum of the hemoglobin spline interpolation is greater than 1.75 g/dL.
- Criterion #2 is fulfilled if the number of time grid points for which the first derivative of the hemoglobin spline interpolation is larger than 0.1 g/dL/week is more than 60% of the total number of time grid points of the interpolated time series.

Anemia therapy assistance software

The anemia therapy assistance software calculates the ESA dose to attain a hemoglobin target of 10.5 g/dl, the mid-point of the target range.

The software supports health care professionals (HCP) to manage anemia in hemodialysis patients. It computes individualized ESA dose recommendations every fourteen days. The software consists of several parts:

1. A physiology-based model of erythropoiesis [1].
   The model comprehensively describes the production of erythrocytes, spanning from stem cells committing to the erythroid lineage, burst-forming unit cells, colony-forming unit cells, erythroblasts and bone marrow reticulocytes to the red cells circulating in the blood stream. It mathematically describes the proliferation, apoptosis and differentiation of the various

cell types and the influence of ESAs and endogenous erythropoietin on these processes. It also covers neocytolysis, a phenomenon recognized to contribute to renal anemia [2 3]. Further, the pharmacokinetics of the endogenous erythropoietin and exogenous ESAs are described with a pharmacokinetic model.

2. A module that adapts the physiology-based model to individual patients [4]. Demographic data (sex, height, post-hemodialysis weight) is used to estimate the euvolemic blood volume using the Nadler formula [5] and to calibrate the number of stem cells committing to the erythroid lineage. A global optimization strategy estimates physiological key characteristics of anemia from recent clinical data of the patient (hemoglobin levels and ESA doses). These biological key characteristics include the red blood cell life span, endogenous erythropoietin production, ESA half-life, ESA dependent apoptosis rate of erythrocyte progenitor cells, and the ESA dependent maturation function of erythrocyte precursor cells. Individualized patient models need to satisfy a set of quality criteria, which include that the mean absolute percentage error between hemoglobin data and model output (i.e. simulated hemoglobin values) is less than 5.5%.
3. A model predictive controller that calculates the next recommended ESA doses [6]. In general, several possible physiological states (i.e. sets of model parameters) are compatible with the patient's retrospective clinical data. Hence, a set of individualized models is used to describe a patient instead of only a single model. A multi-stage model predictive control with a robust horizon strategy for the next recommended dose was designed to incorporate the information of multiple models. An open-loop problem is formulated and repeatedly solved for the set of personalized models for the upcoming sixteen weeks. The model predictive controller determines a single ESA dose for the next recommended dose for all considered patient models such that the predicted hemoglobin curves stabilize within the hemoglobin target range over the next eight weeks. Note, only the next recommended dose is unambiguous. Subsequent doses may vary between different patient models. Predictions are evaluated for their quality; defined by the difference in the predictions of hemoglobin outcomes and differences in dosing strategies between the different patient models and the area outside the target range.

Supplemental Table 2. Clinic names and locations of clinics that participated in the randomized controlled trial.

Clinic Name	Address	Country
**Upper East Side Dialysis Center**	321 East 62nd Street, New York, NY 10065	USA

**Irving Place Dialysis Center**	120 East 16th Street, New York, NY 10003	USA
**St. Raphael Dialysis Center**	137 Water Street, New Haven, CT 06511	USA
**Shoreline Dialysis Center**	34 East Industrial Road, Branford, CT 06405	USA
**Newport Mesa Dialysis Center**	1175 Baker Street, Building B, Costa Mesa, CA 92626	USA

Supplemental analysis

Additional analysis of the primary and secondary outcomes

Supplemental Table 3. Primary and secondary outcome measures (patients that remained in the study ≥90 days). *Data are presented as median (interquartile range). Bootstrapping was used to calculate the median difference and the 95% confidence interval of the difference. Group comparisons were performed by Wilcoxon rank-sum and Chi-squared test as appropriate. ESA, erythropoiesis stimulating agent*

Characteristic	*Intervention group, n=44*	*Standard-of-care group, n=43*	*Median Difference (95% CI)*	*p-value*
Primary outcome measure				
Hemoglobin values in target, %	48 (40, 59)	38 (32,47)	10 (3, 16);	0.004
Secondary outcome measures				
Patients with fluctuating hemoglobin levels, %	45	81	-34 (-52, -13)	0.001
Mean hemoglobin, g/dl	10.4 (10.3, 10.7)	10.7 (10.4, 11.0)	-0.3 (-0.4, -0.01)	0.03
Hemoglobin standard deviation, g/dl	0.7 (0.6, 0.9)	0.9 (0.7, 1.1)	-0.2 (-0.3, -0.04);	<0.001
Hemoglobin distance to target, g/dl)	0.3 (0.2, 0.4)	0.4 (0.3, 0.7)	-0.2 (-0.3, -0.06)	<0.001
Mean monthly ESA dose, mcg/30 days	69 (47, 114)	91 (60, 148)	-23 (-67, 4)	0.06

Mean ESA dose, mcg/30 days/kg	1.1 (0.6, 1.5)	1.5 (0.9, 2.2)	-0.4 (-0.8, 0.1)	0.03

Supplemental Table 4. Primary and secondary outcome measures (patients that completed the study). *Data are presented as median (interquartile range). Bootstrapping was used to calculate the median difference and the 95% confidence interval of the difference. Group comparisons were performed by Wilcoxon rank-sum and Chi-squared test as appropriate. ESA, erythropoiesis stimulating agent.*

Characteristic	*Intervention group, n=40*	*Standard-of-care group, n=41*	*Median Difference (95% CI)*	*p-value*
Primary outcome measure				
Hemoglobin values in target, %	47 (40, 59)	38 (32, 47)	9 (3, 15)	0.008
Secondary outcome measures				
Patients with fluctuating hemoglobin levels, %	45	83	-35 (-55, -16)	<0.001
Mean hemoglobin, g/dl	10.5 (10.3, 10.7)	10.7 (10.4, 10.9)	-0.2 (-0.4, -0.02)	0.07
Hemoglobin standard deviation, g/dl	0.7 (0.6, 0.9)	0.9 (0.7, 1.1)	-0.2 (-0.3, -0.03)	<0.001
Hemoglobin distance to target, g/dl	0.3 (0.2, 0.4)	0.4 (0.3, 0.7)	0.1 (-0.2, -0.06)	<0.001
Mean monthly ESA dose, mcg/30 days	69 (46, 120)	91 (60, 148)	-23 (-63, 6)	0.10
Mean ESA dose, mcg/30 days/kg	1.1 (0.6, 1.5)	1.5 (0.9, 2.2)	-0.4 (-0.8,0.1)	0.04

Supplemental Table 5 Adverse and serious adverse events.

	Intervention group	Standard of care group	Total count (%)
Adverse events	4	1	5 (9.6%)
Serious adverse events	20	27	47 (90.4%)
Total count (%)	24 (46.2%)	28 (53.8%)	52 (100%)
**Category**	**Intervention group**	**Standard of care group**	**Total count (%)**
Access related	2	5	7 (13.5%)
Cardiovascular	3	4	7 (13.5%)
COVID-19	3	2	5 (9.6%)
Fluid overload	3	1	4 (7.7%)
GI related	3	5	8 (15.4%)
Infection	6	4	10 (19.2%)
Musculoskeletal	1	4	5 (9.6%)
Neuropsychiatric	3	0	3 (5.8%)
Pulmonary	0	2	2 (3.8%)
Other	0	1	1 (1.9%)
Total count (%)	24 (46.2%)	28 (53.8%)	52 (100%)

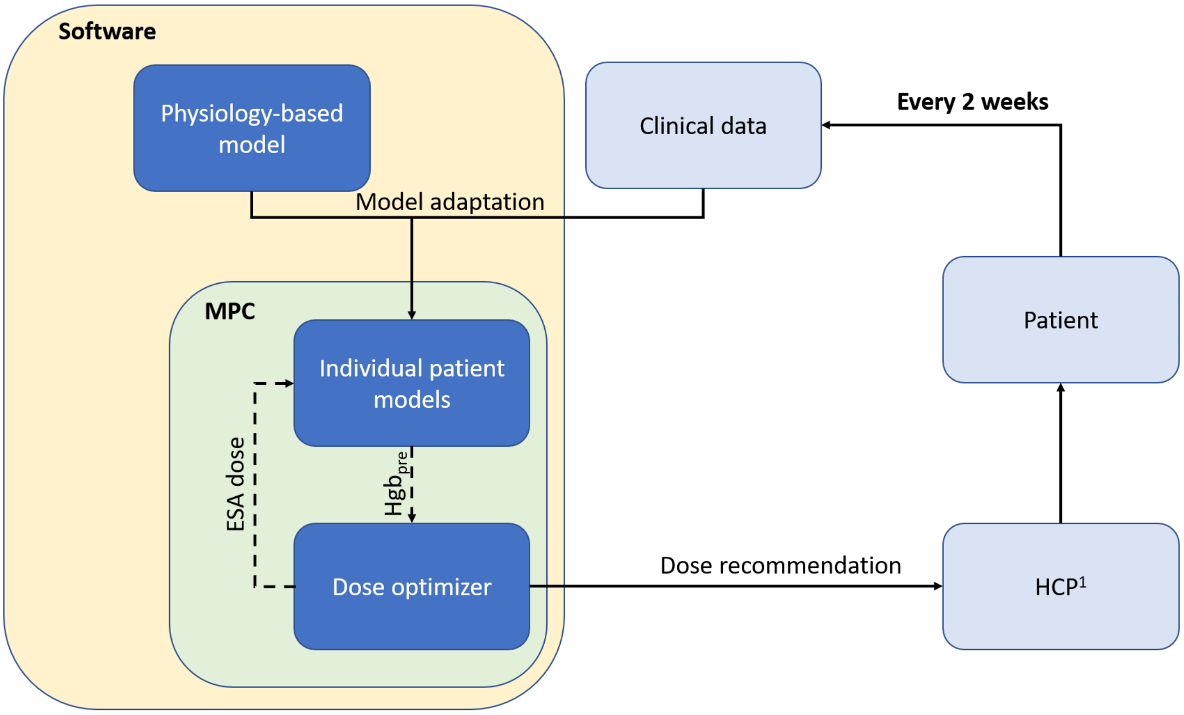

Supplemental Figure 2 Schematic of the anemia therapy assistance system. *The software consists of two components: a physiology-based mathematical model of erythropoiesis and erythrocyte dynamics that is adapted to individual patients and a model predictive controller (MPC) to compute optimal ESA doses based on the model's predictions.* [1]*HCP: health care professional.*

References

1. Fuertinger DH, Kappel F, Thijssen S, Levin NW, Kotanko P. A model of erythropoiesis in adults with sufficient iron availability. J Math Biol 2013;**66**(6):1209-40 doi: 10.1007/s00285-012-0530-0[published Online First: Epub Date]|.
2. Rice L, Alfrey CP. The negative regulation of red cell mass by neocytolysis: physiologic and pathophysiologic manifestations. Cell Physiol Biochem 2005;**15**(6):245-50 doi: 10.1159/000087234[published Online First: Epub Date]|.
3. Rice L, Alfrey CP, Driscoll T, Whitley CE, Hachey DL, Suki W. Neocytolysis contributes to the anemia of renal disease. Am J Kidney Dis 1999;**33**(1):59-62 doi: 10.1016/s0272-6386(99)70258-1[published Online First: Epub Date]|.
4. Fuertinger DH, Kappel F, Zhang H, Thijssen S, Kotanko P. Prediction of hemoglobin levels in individual hemodialysis patients by means of a mathematical model of erythropoiesis. PLoS One 2018;**13**(4):e0195918 doi: 10.1371/journal.pone.0195918[published Online First: Epub Date]|.
5. Nadler SB, Hidalgo JH, Bloch T. Prediction of blood volume in normal human adults. Surgery 1962;**51**(2):224-32
6. Rogg S, Fuertinger DH, Volkwein S, Kappel F, Kotanko P. Optimal EPO dosing in hemodialysis patients using a non-linear model predictive control approach. J Math Biol 2019;**79**(6-7):2281-313 doi: 10.1007/s00285-019-01429-1[published Online First: Epub Date]|.
